# Response Flexibility: The Role of the Lateral Habenula

**DOI:** 10.3389/fnbeh.2022.852235

**Published:** 2022-04-04

**Authors:** Victoria I. Hones, Sheri J. Y. Mizumori

**Affiliations:** ^1^Department of Psychology, University of Washington, Seattle, WA, United States; ^2^Graduate Program in Neuroscience, University of Washington, Seattle, WA, United States

**Keywords:** behavioral adaptation, lateral habenula, motivation, context memory, hippocampus, medial prefrontal cortex

## Abstract

The ability to make appropriate decisions that result in an optimal outcome is critical for survival. This process involves assessing the environment as well as integrating prior knowledge about the environment with information about one’s current internal state. There are many neural structures that play critical roles in mediating these processes, but it is not yet known how such information coalesces to influence behavioral output. The lateral habenula (LHb) has often been cited as a structure critical for adaptive and flexible responding when environmental contexts and internal state changes. A challenge, however, has been understanding how LHb promotes response flexibility. In this review, we hypothesize that the LHb enables flexible responding following the integration of context memory and internal state information by signaling downstream brainstem structures known to drive hippocampal theta. In this way, animals respond more flexibly in a task situation not because the LHb selects a particular action, but rather because LHb enhances a hippocampal neural state that is often associated with greater attention, arousal, and exploration. In freely navigating animals, these are essential conditions that are needed to discover and implement appropriate alternative choices and behaviors. As a corollary to our hypothesis, we describe short- and intermediate-term functions of the LHb. Finally, we discuss the effects on the behavior of LHb dysfunction in short- and intermediate-timescales, and then suggest that new therapies may act on the LHb to alleviate the behavioral impairments following long-term LHb disruption.

## Introduction

One’s ability to behave intentionally, especially when presented with options, involves a number of complex processes such as selectively attending to relevant sensory input, determining whether environmental context conditions have changed from what is expected based on past experience, selecting an appropriate action, assessing the outcome of the selected action relative to internal state information, and then updating one’s knowledge about the context and response outcomes to be prepared for the next encounter. A common driver of all of these processes is not only one’s memory but also the ability of information about one’s internal state to modulate the efficiency of memory processing and thus memory’s impact on subsequent behaviors. Therefore, in order to fully understand real world goal-directed and flexible behavioral adaptation, it is necessary to understand not only how the brain processes new information to form memories, but it is essential to clarify how internal state (i.e., motivation) information comes to regulate the behavioral implementation of context memories.

There are a number of excellent and detailed reviews on brain mechanisms of context memory (e.g., Burgess et al., 2002; Eichenbaum, 2017; Lisman et al., 2017; Maurer and Nadel, 2021). Since the hippocampus (HPC) is known to be critical for context memory, and since hippocampal neurons are sensitive to a broad range of external and internal sensory information (including rewards and aversive stimuli), our definition of “context” extends beyond the external sensory environment. While we have made exciting and significant advances in our understanding of the molecular, as well as neural circuit and systems changes during context learning and memory, precisely how context memory intersects with information about one’s current motivational state to promote adaptive behavioral outcomes is not clear. This is an important problem to solve for it applies to many of our everyday behaviors and decisions. As an example, while you may have learned about and understand the health benefits of exercise, your motivational state may conflict with this knowledge, resulting in you deciding not to go to the gym.

In this review, we focus specifically on the issue of how brain systems that integrate context memory and motivation information come to enable freely-navigating animals to quickly switch behaviors by flexibly responding to changes in environmental conditions. One approach to resolving this issue is to consider the advantage that cortical evolution may have conferred onto a pre-existing, evolutionarily conserved experience-dependent response flexibility brain system that involves the epithalamic structure, the habenula. In fish, amphibians, reptiles, and birds, the habenula receives sensory and internal state information while sending strong signals to midbrain structures that regulate overt actions (Stephenson-Jones et al., 2012). A critical role of the habenula in response flexibility is supported by findings that habenula disruption leads to impaired approach or avoidance responses to dynamic shifts in internal conditions or information, such as hormone levels (Ogawa et al., 2021), rules for task performance (Palumbo et al., 2020), or external environmental context cues. As the neocortex evolved in mammals, so did the prominence of the lateral division of the habenula (the lateral habenula, or LHb) as well as LHb connectivity with frontal cortical areas. Thus, it has been hypothesized that while the mammalian LHb continues to support flexible responding, it does so based not only on sensory and internal state input but also the frontal cortical (presumably context memory) input (Baker and Mizumori, 2017; Mizumori and Baker, 2017). Given the growing number of excellent and relevant published review articles, in the following sections, we only briefly highlight key findings that support the claim that the LHb plays important roles in context memory, processing motivation information, integrating context memory and motivational state information, and in response flexibility. Then we suggest a novel hypothesis to address the unanswered question of how the LHb enables adaptive context-dependent response flexibility.

## LHb Is A Hub for Memory and Internal State Information

At any given point in time, the current constellation of neural activity and functional connectivity across the brain (considered here as the *internal state*) defines the neural foundation within which new information is processed. One often refers to such foundational neural states relative to a particular functional attribute such as a cognitive state, motivational state, and/or behavioral/response state. As an example, the patterns of cortical neural activity that exist prior to stimulus exposure (reflecting the cognitive state) effectively determine how new sensory information is processed and perceived, which in turn defines our interpretation and interactions with the world. In everyday life, different neural states do not function independently, but rather they are interdependent. For example, it is well known that altered motivational states, such as that which occurs when stressed, can bias the efficiency of information processing to improve (or impair) memory. The LHb presumably at least identifies, if not also retains (Andalman et al., 2019), current cognitive and motivational state information in order to adjust behavioral responding as task conditions change.

### Cognitive State: Context Memory

Significant evidence supports the generally-accepted view that the HPC is critically important for context memories (e.g., Burgess et al., 2002; Howard and Eichenbaum, 2013; Smith et al., 2013; Eichenbaum, 2014; Place et al., 2016; Maurer and Nadel, 2021). Many studies have shown that place cells in the HPC fire strongly when an animal occupies a particular location (a place field; O’Keefe and Nadel, 1978; Gothard et al., 1996). When almost any feature of the context changes, HPC place fields remap or change their spatial and temporal patterns of firing. Additionally, place fields link to represent recent past, present, and future context information (e.g., Muller and Kubie, 1987; Wilson and McNaughton, 1993; Mizumori et al., 1999; Ferbinteanu and Shapiro, 2003; Leutgeb J. K. et al., 2005; Leutgeb S. et al., 2005; Smith and Mizumori, 2006; Nakashiba et al., 2009). In the natural world, it would be unfavorable for an animal to explore an environment similar to one that they have explored in the past that led to danger. Thus, when faced with a new but similar context, the HPC is thought to retrieve information about similar previous experiences (Spiers et al., 2015), then evaluate the extent to which the current context varies from the retrieved (expected) context (Mizumori et al., 1999; Vinogradova, 2001). The degree of similarity between expected and current context information seems reflected in HPC output signals (Mizumori, 2013). Without proper functioning of the HPC, context memory-guided behavior becomes significantly impaired (e.g., Morris et al., 1982; Bradfield et al., 2020; Gridchyn et al., 2020).

#### LHb Plays a Role in Context Memory

Early studies that probed LHb functions discovered that its inactivation results in analgesic-like effects at the time of tonic pain presentation (Fuchs and Cox, 1993). Soon after, it was shown that electrical stimulation of the LHb resulted in aversive behaviors perhaps by generating an aversive signal (Matsumoto and Hikosaka, 2009a; Friedman et al., 2011). Indeed, LHb terminals in the ventral tegmental area (VTA) and rostromedial tegmentum (RMTg) mediate behavioral avoidance (Lammel et al., 2012; Stamatakis and Stuber, 2012; Brown and Shepard, 2013). Therefore, LHb is often considered the “aversion center” of the brain (Baker et al., 2016). As a result of more recent studies, however, a number of functions are now attributed to the LHb in addition to the initial idea that it serves to provide an aversive signal. For instance, Congiu et al. (2019) found that aversive foot shocks not only excite the majority of LHb neurons, but also inhibit the activity of a small population of excitatory LHb neurons, indicating a more complex function of the structure. Also, neurons in the LHb have been found to exhibit changes in activity patterns to rewards, suggesting the LHb contributes to signaling information to *both* aversive and rewarding stimuli (Matsumoto and Hikosaka, 2009a). An important role of the LHb in HPC-related context memory has been suggested in numerous studies. For example, LHb inactivation results in disruption of memory retrieval as well as an inability to update the spatial configuration of the environment (Mathis et al., 2015). LHb neurons have also been shown to keep track of choice outcomes, implicating it in memory for decisions made during goal-directed tasks (Baker et al., 2015; Kawai et al., 2015). LHb, then, appears to participate in the signaling of memories in the recent past, as well as memories collected over longer periods, suggesting a perhaps more general modulatory role in memory (Bromberg-Martin et al., 2010b).

Numerous studies more explicitly show that the LHb is necessary for accurate context memory processing since its disruption impairs HPC-dependent context memory tasks such as the spatial delayed alternation and probabilistic reversal maze tasks (Baker et al., 2015; Barker et al., 2017; Baker et al., 2019). LHb is also involved in tasks such as novel object recognition (Goutagny et al., 2013) and the water maze task (Thornton and Davies, 1991; Lecourtier et al., 2004). It has also been shown that the spiking of LHb neurons aligns with the HPC theta phase, suggesting a crucial interaction between the two regions (Aizawa et al., 2013). In the case of context-dependent fear memory, which necessitates the association between a context and an aversive cue presentation, inactivation of the LHb impaired the ability to appropriately respond to an aversive context (Durieux et al., 2020). In sum, a role for the LHb in context memory seems clear.

#### What Is the Source of Context Information for the LHb?

An important outstanding question is the source of context information for the LHb. It is possible that the HPC relays contextual information to the LHb to guide proper behavioral responses. There are, however, no known direct connections between the HPC and the LHb, suggesting the involvement of an intermediary brain region. Many studies have demonstrated the importance of the communication between the cortex and HPC in context memory and response flexibility (Spellman et al., 2015; Tamura et al., 2017; Avigan et al., 2020). Notably, one cortical brain region that plays a crucial role in action selection when responding to changing contexts, as well as receiving strong synaptic innervation from the HPC, is the medial prefrontal cortex (mPFC; Gilmartin and Helmstetter, 2010; Brockett et al., 2020). When the infralimbic and prelimbic areas of the mPFC are inactivated, rats exhibit a significantly higher escape latency if the escape platform in a water maze is shifted to a different area of the maze compared to controls (de Bruin et al., 1994; Haddon and Killcross, 2011). Some mPFC neurons are preferentially active during specific behavioral states, giving rise to possible state information that the LHb can use to influence behavior (Halladay and Blair, 2015). Dysfunction of either the HPC or the mPFC often results in similar context memory and decision-making impairments, but several studies have attempted to disentangle their possible unique properties (Corcoran and Quirk, 2007). The HPC plays a greater role in the formation and retrieval of memories about spatial contexts (O’Keefe and Nadel, 1978; Maurer and Nadel, 2021), while the mPFC plays a more crucial role in the retrieval of distant memories that are generalizable to similar contexts (DeNardo et al., 2019; Samborska et al., 2021).

mPFC neurons display neuronal activity comparable to that of the LHb in that specific subpopulations display different activities in response to appetitive and aversive stimuli (Warden et al., 2012; Rubio et al., 2019; Capuzzo and Floresco, 2020). This suggests that the mPFC and LHb may have common functions and/or they both participate in behavioral flexibility. Such findings imply a potentially important interaction between the mPFC and LHb. Indeed, studies using both anterograde and retrograde tracers have identified mPFC fibers terminating at the LHb, suggesting a functional connection (Kim and Lee, 2012; Mathis et al., 2021). What might be the functions of mPFC-LHb connections? Along with the evolution of the cortex came the ability to have greater intentional control over the execution of behaviors. As the evolution of neocortical memory systems continued, so did the establishment of connectivity between cortical memory areas and subcortical structures that influence action, such as the LHb. mPFC signals in particular, then, become a strong candidate intermediary structure to communicate HPC-derived context and other valence-related information necessary for appropriate and adaptive behavior. Recent studies have attempted to uncover the nature of the signal from the mPFC to the LHb as well as its impact on behavioral output. Mathis et al. (2021) conducted a sequence of experiments characterizing the activity pattern of mPFC neurons that project to the LHb and their role in stress. They found that mPFC cells send signals to the LHb in the presence of a stressful event such as a foot shock. Moreover, the LHb cells that received signals from the mPFC had differential behavioral output based on the different network projection profiles. For instance, the mPFC-LHb-locus coeruleus projection played a major role in cocaine-seeking, implicating this projection in reward-seeking behavior. The mPFC-LHb-raphe projection was implicated in freezing behavior in response to a stressor. Interestingly, these findings are consistent with the theory that the mPFC relays context-specific information to the LHb, which can serve as a brake signal (Sleezer et al., 2021) to cease behaviors when appropriate or to engage behaviors in other aversive contexts.

Another potential source of context information for the LHb is the septal complex. The septum is a subcortical midline structure that is divided into medial and lateral septal portions (MS and LS, respectively) each of which have strong connections with the HPC and LHb (Swanson and Cowan, 1979). Inactivation of the MS results in spatial working memory deficits, impairments in HPC place cell activity, as well as impairments in processing contextual information, implicating the septum in processing contextual memory (Mizumori et al., 1989b; Leutgeb and Mizumori, 1999; McGlinchey and Aston-Jones, 2018). Separating the functional contributions of the LS and MS to LHb has been challenging. The LS receives input primarily from the HPC, and projects to the MS, which projects back to the HPC *via* the fornix. A strong output of both the MS and LS is to the LHb (for an in-depth review of septal inputs and outputs, see Swanson and Cowan, 1979). Silencing either the LS or the MS results in the overall decrease in avoidance behavior, while stimulation of excitatory MS inputs to the LHb induced conditioned place aversion in a two-chamber avoidance task (Veening et al., 2009; Zhang et al., 2018). The aversion-induced behavioral effect was positively correlated with stimulation frequency, which suggests that MS inputs play a significant role in driving aversive behavior in response to an aversive context. In terms of septal influences on context memory, the MS and LS are thought to play a role in maintaining HPC theta that is necessary for proper spatial and contextual memory integration (Tsanov, 2018). While the MS has reciprocal connections with the HPC, the LS only receives unidirectional inputs from the HPC (Tsanov, 2018). Thus, the MS may be driving both LHb and HPC activity necessary for contextual information processing, while the LS may be filtering contextual HPC information and relaying this signal to the LHb for proper adaptive behavioral output (Yetnikoff et al., 2015; Tsanov, 2018; Wirtshafter and Wilson, 2019).

### Motivational State

The primary motivating factor of a living organism is the need for survival. Thus, an animal’s experiences and actions in the world are highly influenced by recent previous experiences and motivations. For instance, an animal’s willingness to work for food is influenced by whether or not they have eaten recently. There are many outstanding reviews on the neural circuitry underlying motivational systems (e.g., Berridge, 2004; Bromberg-Martin et al., 2010a; Morales and Margolis, 2017; Petrovich, 2018; Burdakov and Peleg-Raibstein, 2020). The distinct and overlapping motivational systems contribute to a specific profile of a motivational state. Motivational systems research has typically focused on relating one’s internal state to specific biologically-motivated behaviors such as hunger and feeding behaviors, as well as reproductive hormones and mate-seeking behaviors. Recent theories postulate that motivation structures such as the lateral hypothalamus serve as an interface between motivation and cognition systems (e.g., Petrovich, 2018; Burdakov and Peleg-Raibstein, 2020), but it is not yet known how motivational state influences the type of response flexibility needed for accurate goal-directed and context-dependent navigation.

#### Motivational Information Is Processed by the LHb

The LHb is known to receive a wide range of inputs relating to one’s internal and external motivational state, such as value-based signals (Bianco and Wilson, 2009; Trusel et al., 2019), gustatory signals (Stamatakis et al., 2016), and circadian rhythm signals (Baño-Otálora and Piggins, 2017). For illustrative purposes, below we focus on only a few direct sources of motivation information that are known to modulate LHb activity, and consequently response flexibility.

The entopeduncular nucleus (EPN) provides significant motivational input to the LHb. Formerly thought to be primarily involved in the motor movement (Hauber, 1998), LHb lesions often resulted in cognitive and not motor-related deficits (Miller et al., 2006). Additional research unearthed another potential role for the EPN, implicating it in reward valuation (Hikosaka et al., 2006). Hong et al. (2011) found antidromic LHb signals in the globus pallidus (GP), a primate EPN analog, that differentially responds to reward. Bilateral inactivation of the GP led to the inability to learn new associations and task contingencies, implicating the GP in adaptive behavior (Piron et al., 2016). Interestingly, the EPN exhibits graded levels of firing activity corresponding to the expectation of an outcome, suggesting that the EPN encodes the value of an action as well as the outcome (Stephenson-Jones et al., 2016). These reward-related signals are sufficient to drive motivation. For example, Cerniauskas et al. (2019) found that most EPN neurons synapse onto VTA-projecting LHb neurons, driving LHb hyperexcitability and inducing motivational impairments. Thus, the EPN appears to communicate reward-related signals to the LHb, as well as contributes to driving motivation.

The lateral hypothalamus (LH) also provides behaviorally-relevant motivational information to the LHb.regulating anxiety and depressive-like behaviors. The LH is functionally heterogeneous, containing both glutamatergic and GABAergic neurons, activation of which results in different behavioral responses (Jennings et al., 2013; Trusel et al., 2019). The LH has prominent projections that terminate in the LHb, and stimulating this projection results in both excitatory and inhibitory responses in the LHb, suggesting a potential bidirectional influence of LH on LHb activity (Stamatakis et al., 2016). In another study, orexinergic LH signals that terminate in the LHb result in LHb inhibitory responses, as well as increases in aggressive behavior (Flanigan et al., 2020). Excitatory responses in the LHb as a result of glutamatergic LH stimulation result in aversive behavior and it plays a role in generating a prediction signal for future negative events (Lecca et al., 2017; Lazaridis et al., 2019). As such, the LH exhibits a two-factor influence on LHb activity and subsequent motivated behavior.

The lateral preoptic area (LPO) is a critical structure for motivational drive and it provides one of the largest inputs to the LHb (Yetnikoff et al., 2015). Comparable to other structures that bidirectionally influence LHb activity, the LPO also exerts bivalent control over the LHb, which influences the motivational state. Interestingly, glutamatergic and GABAergic LPO neurons simultaneously synapse on individual LHb neurons and both are activated by aversive stimuli (Barker et al., 2017). However, when stimulated individually, glutamatergic and GABAergic LPO inputs to the LHb produce divergent behavioral responses. This suggests that LPO activity is able to influence individual LHb neurons and drive opposing motivational states.

All mammals have a biological clock that regulates the activity of physiological functions (i.e., immune system coordination) and prepares them for specific motivated behaviors. The circadian rhythm is influenced by both intrinsic (i.e., physiological states, autonomic arousal) as well as extrinsic activity (i.e., daylight, food). For instance, animals that exhibit diurnal rhythms, like most primates, have increased motivation during the day to socialize and hunt for food. Without regular circadian rhythmicity, motivation lowers and animals tend to make less-optimal decisions (Acosta et al., 2020). Although many brain regions have been shown to play a role in this internal rhythmicity, the suprachiasmatic nucleus (SCN) is the most prominent. The SCN organizes the activity in the brain that inevitably influences the body through its inherent ability to oscillate and synchronize the activity of multiple brain regions. The LHb appears to play an important role in circadian rhythmicity (Sakhi et al., 2014; Baño-Otálora and Piggins, 2017; Mendoza, 2017), likely as a result of receiving significant input from the SCN. Paul et al. (2011) found that LHb lesions resulted in motor and circadian rhythm impairment, implicating the LHb in the relay of SCN circadian rhythmicity important for behavior. It is possible that the LHb is integrating information about circadian rhythms to appropriately time-motivated behaviors for optimal decisions (Mendoza, 2017). Lastly, reward-signaling structures (i.e., the VTA) have shown to exhibit circadian rhythm firing, highlighting the LHb’s circadian rhythm regulation of reward systems more generally (Bussi et al., 2014).

While the EPN, LH, LPO, and the SCN each strongly and directly relays motivational information to the LHb, it is worth noting that motivation information may bias the nature of information arriving in LHb from other structures not traditionally considered to be related to motivation, such as the mPFC. For example, in times of deliberation, an animal must evaluate options and select an action that would lead to the most optimal outcome. These actions most often have to do with approaching reward or avoidance of punishment. Selecting the action with the most optimal outcome involves evaluating similar previous actions and their respective outcomes. This manifests itself in the form of reward and reward prediction error signals (Bromberg-Martin and Hikosaka, 2011). Such value-based (motivation-related) signals are encoded at the time of action selection and outcome evaluation and are used to inform future behavior. The VTA and nucleus accumbens (NAcc) are likely involved early in this decision process since they seem to track outcomes and generate reward prediction signals as shown by neural activity that correlates with behavior and motivational effort for both the VTA (Bromberg-Martin et al., 2010a) and NAcc (Hamid et al., 2016). NAcc activity, however, is not dependent on VTA input (Floresco et al., 1998). Instead, the basolateral amygdala (BLA), involved in reward-related associative learning, appears to drive NAcc reward firing. Importantly, these NAcc signals come to influence LHb activity which then drives downstream structures, such as the VTA, toward the facilitation of reward approach or punishment avoidance behaviors (Bianco and Wilson, 2009). It is unclear whether the direct projection from the NAcc to the LHb influences motivated behavior.

The Ventral Pallidum (VP) receives significant reward-related signals from the NAcc and it in turn relays this information to the LHb *via* both glutamatergic and GABAergic projections (Soares-Cunha et al., 2020; Stephenson-Jones et al., 2020). Inhibition of excitatory VP inputs to the LHb abolished reward-seeking behavior, while inhibition of inhibitory VP inputs to the LHb abolished behavioral avoidance (Knowland et al., 2017; Stephenson-Jones et al., 2020). Therefore, subpopulations of VP neurons (perhaps influenced by the NAcc) bi-directionally drive behavior *via* their inputs to the LHb in different motivational contexts.

The VTA-PFC projection has been implicated in many cognitive and behavioral processes such as mood regulation (Walsh and Han, 2014). Importantly, stimulation of VTA neurons induces neuroplastic strengthening of cortical inhibitory circuits, thereby inhibiting overall PFC activity (Zhong et al., 2020). Concurrently, dopamine injection increases theta coherence between the HPC and mPFC (Benchenane et al., 2010). It is possible that reward-related dopaminergic signals reach the PFC to update cortical information such that it more precisely represents the present situation relative to the HPC. In this way, information sent from the mPFC to the LHb is behaviorally relevant, thereby promoting timely and appropriate behaviors.

#### Motivational Influence on Contextual Information

Behaviorally, many experiments have exemplified the positive influence of motivation, induced by factors such as reward magnitude, on performance in context-related goal-oriented tasks (Sänger and Wascher, 2011). Additionally, the presence of reward in specific predictable locations results in animals returning to these rewarded locations at a higher rate in the future (see Anselme, 2021 for review). It is possible that context information within PFC signals and motivational information from a number of subcortical structures arrive at LHb in a temporally precise manner that biases LHb outputs appropriate for a particular context. For example, Chang et al. (1998) showed that a subset (~5%) of PFC and NAcc neurons respond to a rewarding stimulus simultaneously. At the population level, theta oscillatory synchrony between the PFC and VTA increases during actions that were likely to result in a reward (Park and Moghaddam, 2017). The synchronized theta activity may be linking the PFC contextual information with the VTA reward signal, allowing the LHb to receive in a timely manner input to associate motivational and contextual PFC information.

The mPFC is thought to serve as an inhibitory control for motivated behaviors by interacting with the HPC and retrieving context-appropriate memories to inform future action selection (McDonald et al., 2008; Chen et al., 2013; Zelikowsky et al., 2013; Porter et al., 2019). Interestingly, HPC neurons that project to the mPFC (Hsu et al., 2018), and mPFC neurons that project to the LHb (Mathis et al., 2021), appear to inhibit motivated behavior to seek reward, implicating this circuit in the motivational regulation of reward seeking. The HPC itself has been shown to monitor and respond to motivational states when associated with a particular context (Kennedy and Shapiro, 2009). Specifically, Kennedy and Shapiro (2009) show that HPC single units respond preferentially to a context paired with reward only when the rats were hungry or thirsty. This may be a result of motivational inputs directly influencing the HPC, as many of the aforementioned motivational circuits, such as the VTA (Gasbarri et al., 1994; Martig et al., 2009; Ghanbarian and Motamedi, 2013), SCN (Phan et al., 2011; McCauley et al., 2020), LH (Samerphob et al., 2015; Noble et al., 2019; Rezaee et al., 2020), EPN (Sabatino et al., 1986; Chen Y. et al., 2020), and amygdala (Sheth et al., 2008; Tsoory et al., 2008; Ghosh et al., 2013), synapse onto and influence HPC activity. In sum, these findings demonstrated that the LHb is not alone in integrating motivational and memory information since cortical memory information likely already incorporates some aspects of motivation. What distinguishes the LHb from cortical systems that may be influenced by motivational and memory states is that LHb output may more directly determine optimal and adaptive behavior.

#### Motivation and Memory-Guided Decisions

All previous experiences serve as roadmaps for future decisions, actions, and their respective outcomes. The selection of an action that leads to a particular outcome will occur only when an animal is motivated. As mentioned above, the experience of hunger/satiation, the state of the biological clock, and internal valuation of possible outcomes define one’s motivational states which guide and direct goals. As such, there is a necessary link between motivations and decisions, where highly motivated animals exhibit more effortful behavior in order to obtain a reward. There are numerous studies examining the effect of motivational states on decision making, where animals must choose between small or large rewards that necessitate large or small amounts of effort, respectively (Floresco and Ghods-Sharifi, 2007; Mai et al., 2012). Animals will exert effort to seek reward up to a certain point until the effort required is too great and no longer worth the payoff. This threshold is influenced by internal state and motivation, often changing depending on context (Knauss et al., 2020). Past experiences, too, shape internal state and motivation (Dysvik and Kuvaas, 2013). As animals evoke memories of similar previous experiences to evaluate the current context and most optimal choice, the animal’s associations with a previous choice will influence their motivation and, subsequently, their decisions and actions. As a result of the functional interactions between the LHb and mPFC, the LHb serves as an integrative node for motivational and contextual information for the purpose of ensuring adaptive and flexible responses.

## LHb and Response Or Choice Flexibility

Regardless of the species under study, it is often suggested that the habenula regulates an animal’s ability to switch learned behavioral and cognitive strategies when a goal or context changes. This switch is likely, not due to successive learning by different memory systems since strategy switching occurs much more quickly than new learning, and since multiple memory systems are thought to essentially operate in parallel (e.g., Mizumori et al., 2004; White et al., 2013; Hasson et al., 2015). The ability to rapidly change behavioral strategies is often attributed to the mPFC (e.g., Dalley et al., 2004; Ragozzino, 2007), but species without a defined prefrontal cortex (e.g., fish; Agetsuma et al., 2010; Okamoto et al., 2012; Stephenson-Jones et al., 2016) show remarkable abilities to flexibly respond in adaptive ways when a change in either the external sensory environment (including social cues, Chou et al., 2016) or internal state (such as motivation or use of learned task rules; Parker et al., 2012; Randlett et al., 2015; Cherng et al., 2020; Palumbo et al., 2020) occurs. Fish habenula, as an example, is often suggested to enable response or behavioral flexibility by integrating the different types of information (including the evaluation of response outcomes, motivation state, and sensory cues) needed to strategically switch behavioral responses/strategies in simple and more cognitively demanding tasks.

The mammalian LHb (relative to the MHb) seems to have co-evolved with the cortex to process more complex sensory and memory-related information, and in this way enable more refined and flexible behavioral control when performing cognitive tasks that depend on limbic cortical processing (e.g., by HPC and mPFC; Ichijo and Toyama, 2015; Ichijo et al., 2017; Mizumori and Baker, 2017). Early reports of the effects of lesions on the mammalian LHb showed that rats became unable to switch or maintain learned behaviors when contingencies changed in appetitive, HPC-dependent tasks (Thornton and Evans, 1984; Thornton and Davies, 1991). Importantly, LHb inactivation or lesion do not affect working memory, nonspecific sensory processing, identification of spatial locations, new learning, memory retrieval, motivation, behavioral activation, or reward discrimination *per se* (e.g., Thornton and Evans, 1984; Thornton and Davies, 1991; Lecourtier et al., 2004; Stopper and Floresco, 2014; Baker et al., 2015, 2019; Mathis and Lecourtier, 2017; Mathis et al., 2017). The hypothesis that the LHb importantly contributes to cortically-mediated response flexibility received additional support when rats were tested in HPC and mPFC-dependent tasks for which there is no right or wrong response, but rather choice preferences reveal an animal’s responsiveness to changing task conditions. Such tasks require continuous and subjective value assessments to direct choice responses. LHb inactivation was found to be sufficient to disrupt such choice preferences when the probability of obtaining rewards shifted, or when the delay before reward access varied, during both operant testing (Shohamy et al., 2009; Dickerson et al., 2011; Delgado and Dickerson, 2012; Stopper and Floresco, 2014) and testing on open, elevated mazes (Baker et al., 2015, 2019). Importantly, spatial processing was not required for task performance, suggesting that the HPC involvement was related to other more integrative features of the task structure since changed preferences were observed only after the task structure shifted.

Understanding the specific processes and neural circuitry underlying the transformation between memory/motivational integration and the execution of behaviors is challenging since the LHb is not considered to be part of the motor output pathway that supports specific actions. Rather it is often explained that LHb’s impact on response flexibility relies on its control over structures known to be important for the execution of voluntary actions (raphe nucleus and the VTA; e.g., Baker et al., 2019). Such an explanation is not satisfactory for it is still unclear how LHb-to-raphe or LHb-to-VTA signals can account for the type of response flexibility often attributed to the LHb. It is suggested here that one approach to resolving this apparent dilemma is to take a reverse engineering approach to this question. That is, the following starts by discussing the nature of the information represented by LHb neurons when rats are engaged in an HPC and mPFC-dependent natural foraging task. From there, we consider which of many brain structure(s) may be strategically informed by patterned LHb output to generate or enable flexible responses.

### LHb Neural Representation During Goal-Oriented Free Navigation

If the LHb enables flexible behavioral responses to changing task conditions, one might expect LHb neural activity to somehow reflect this function. Indeed, the LHb has been shown to be a critical part of the neural circuit that generates prediction error signals when task conditions change. The well-known dopamine neural response to prediction errors is driven at least in part by the LHb (Christoph et al., 1986; Matsumoto and Hikosaka, 2007; Ji and Shepard, 2007; Bromberg-Martin and Hikosaka, 2011; Proulx et al., 2014; Baker et al., 2015; Tian and Uchida, 2015; Lalive et al., 2021), even though this occurs indirectly through the rostromedial tegmentum, or RMTg; Li et al., 2019). While these findings illustrate that flexible behavior is likely mediated by more than brain mechanism (e.g., Floresco, 2013), it clearly shows that LHb neural activity is driven by memory-based outcome expectations. Further evidence for the impact of experience on LHb neural responsiveness is the well-documented change in LHb cell firing that occurs during aversive task performance as well as duringstress (e.g., Stamatakis and Stuber, 2012). Is the coding and integration of mnemonic and motivational information sufficient to ensure flexible responding? Recordings of LHb neural activity during a navigation-based foraging task shed new light on this question.

Using a pellet-chasing task that did not require HPC-based context memory, Sharp et al. (2006) described striking velocity-correlated neural activity in rat LHb. A subsequent study by Baker et al. (2015) confirmed the existence of prominent and strong (often *r* > ± 0.85) velocity-correlated LHb neural activity but this time as rats performed an HPC-dependent spatial working memory task. This result was surprising given the generally accepted view that the LHb contributes to learning and memory by signaling aversive/negative events/consequences/information (see review by Baker et al., 2016). However, Baker et al. (2015) also described another group of LHb neurons that responded to reward encounters, the expectation of rewards, and reward prediction errors in manners similar to the responses of primate LHb reward-responsive neurons described by Matsumoto and Hikosaka (2007). Further, about a third of the recorded LHb neurons showed conjunctive coding of reward and velocity information. During subsequent probe trials in which the reward condition or context was unexpectedly altered, the velocity correlate was retained albeit the overall firing rate was lowered. This pattern of reward and context coding by LHb neurons suggests that the LHb tracks the ongoing behavior of animals, but that the strength of movement state signals may be regulated by reward-related information. Perhaps this behavioral tracking feature is related to the recent report that LHb neurons encode a history of experiences (Andalman et al., 2019). A combination of reward and movement state neural signaling has also been reported for an important efferent structure of the LHb, the VTA (Puryear et al., 2010; Jo et al., 2013), and strong movement state information has been described as a major VTA afferent structure, the lateral dorsal tegmentum (LDTg; Redila et al., 2015). LDTg neurons were postulated to regulate reward responses of DA neurons according to the learned behaviors needed to obtain rewards. What might be the function of LHb movement state signals?

To aid in our understanding of the significance of LHb movement-related neural signals for response flexibility, it is helpful to first consider the finding by Aizawa et al. (2013) that the spiking of LHb neurons is preferentially related in time to the peaks of simultaneously recorded HPC theta rhythms. While a strong argument was made that the LHb spike-HPC theta phase coherence resulted from a common input from the vertical limb of the diagonal band of Broca, this explanation does not address the issue of interest here, and that is how might LHb enable task-specific response flexibility. To shed new light on this issue, we offer the following hypothesis:

Hypothesis*: During active navigation, the LHb may track and then relay information about one’s ongoing behaviors to signal at appropriate times when downstream brainstem structures should drive hippocampal theta. In this way, LHb enables flexible responding not because e it selects a particular action, but rather because it enhances a hippocampal neural state that is often associated with greater attention, arousal, and exploration. In freely navigating animals, these are essential conditions that are needed to discover and implement appropriate alternative choices and behaviors*.

The HPC theta rhythm is known to encourage exploratory behaviors, increase arousal and attention, and improve learning and memory (e.g., Winson, 1978; Mizumori et al., 1989b; Leutgeb and Mizumori, 1999; Lega et al., 2012; Buzsáki and Moser, 2013). Although the LHb does not have direct anatomical connections with the HPC, functional coupling between LHb and HPC has been demonstrated since LHb spikes exhibit cortical synchrony with the HPC theta phase (Aizawa et al., 2013). Such spike-phase coherence is thought to reflect periodic influences of one structure on another (Singer, 1993; Engel et al., 2001; Buzsáki, 2002). Recent evidence (described below) suggests that a number of prominent LHb efferent targets in the hindbrain area may serve as important nodes for communication from the LHb and HPC. These structures receive substantial input from the LHb, and they are considered to be critical pacemakers for HPC theta. Thus, spiking activity in the LHb may enable response and cognitive flexibility by regulating the HPC theta state to optimize exploration-related neural and memory plasticity. Such regulation could strengthen and/or maintain ongoing memory operations by the HPC during navigation of familiar contexts, as well as enable increased cognitive and response flexibility when task conditions change.

### Transforming LHb Neural Signals Into Hippocampal Neural States That Support Cognitive and Response Flexibility

**Figure 1:**
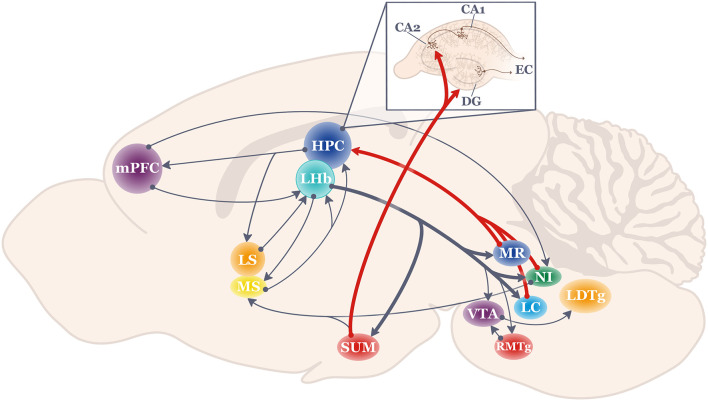
Schematic of the anatomical projections to and from the lateral habenula (LHb; shown in teal). Thick lines highlight LHb projections most strongly related to hippocampal function. HPC, hippocampus; LC, locus coeruleus; LDTg, lateral dorsal tegmentum; LHb, lateral habenula; LS, lateral septum; mPFC, medial prefrontal cortex; MR, medial raphe; MS, medial septum; NI, nucleus incertus; RMTg, rostromedial tegmentum; SUM, supramammillary nucleus; VTA, ventral tegmental area.

The diverse array of LHb efferent targets is well documented (as reviewed in Kim, 2009; Baker et al., 2016; Mizumori and Baker, 2017). Of particular interest here are those that are considered theta-pacemaker structures for the HPC, such as the nucleus incertus, supramammillary nucleus, the median raphe, and the locus coeruleus (Figure 1). In contrast to the traditional view that hindbrain structures regulate slow processes such as the general state of arousal, recent findings demonstrate temporally and spatially-specific regulation of HPC physiology and behavior by these brain regions.

The Nucleus Incertus (NI) lies in the midline periventricular central gray region of the pontine hindbrain, and it is generally considered to be an essential part of the ascending reticular activating system (Steriade and Glenn, 1982) since it innervates structures known to regulate HPC theta such as the MS (Vanderwolf, 1969; Vertes and Kocsis, 1997; Goto et al., 2001; Olucha-Bordonau et al., 2003; Ma et al., 2013). Also, stimulation or inhibition of the NI up- or down-regulates active locomotion (respectively), as well as modulates physiological indices of arousal and HPC theta (Nuñez et al., 2006; Lu et al., 2020). For example, NI neurons preferentially fire at the initial ascending phase of the HPC theta rhythm (Ma et al., 2013), possibly to reset theta (Martínez-Bellver et al., 2017). Evidence that NI impacts on HPC physiology have consequences for HPC-based memory has been demonstrated using a variety of behavioral tasks (Gil-Miravet et al., 2021), including context fear conditioning (Szönyi et al., 2019), various maze-based tasks (Nategh et al., 2015; Albert-Gascó et al., 2017), as well as spatial working memory operant tasks (Albert-Gascó et al., 2017; Garcia-Diaz et al., 2019).

GABA and glutamate incertus neurons (Lein et al., 2007; Cervera-Ferri et al., 2012) express receptors for stress-related hormones (corticotropin-releasing factor, or CRF) and contain multiple neuropeptide markers for stress and arousal such as neuromedin, relaxin, D2 dopamine receptors, and orexin/hypocretin (e.g., Jennes et al., 1982; Kubota et al., 1983; Ma et al., 2013; Lu et al., 2020). Therefore, the NI had been studied primarily for its role in different physiological states. However, as noted above, more recent emerging evidence clearly shows that especially the relaxin-3 NI neurons likely play a significant role in HPC-based memory (Gil-Miravet et al., 2021). For example, these relaxin-3 neurons are the ones that preferentially fire at the initial ascending phase of the HPCtheta rhythm (Ma et al., 2013). Furthermore, Szönyi et al. (2019) identified an incertus-HPCcircuit that may determine which CA1 pyramidal neurons take part in context memory processing: NI long-range GABAergic neurons project directly and indirectly (*via* the medial septum) to HPCsomatostatin neurons to regulate the excitatory/inhibitory balance in stratum oriens of CA1, thereby helping to select which cells participate in memory networks and which ones do not. Since the NI receives strong projections from the prefrontal cortex and the LHb (Goto et al., 2001; Lu et al., 2020), one or both of these incertus afferent systems may drive the NI to regulate HPC neural activity in context-specific ways.

The supramammillary nucleus (SUM) is another deep brain structure of interest when considering how LHb neural activity may translate to HPC-mediated response flexibility. The SUM is a hypothalamic structure that provides strong direct and indirect (*via* the medial septum) theta-rhythmic inputs to the HPC (Haglund et al., 1984; Vertes, 1992, 2015; Kirk and McNaughton, 1993; McNaughton et al., 1995; Kocsis and Vertes, 1997; Vertes and McKenna, 2000; Ito et al., 2018). The overall functional impact of the SUM input is to not only generate HPC theta (Pan and McNaughton, 2004), but SUM afferents amplify neocortical input to the HPC by increasing the responsiveness of dentate gyrus granule cells to input from the entorhinal cortex through disinhibition of inhibitory interneurons (Mizumori et al., 1989a). Such regulation of the excitatory state of granule cells may selectively promote information arriving from the entorhinal cortex. More recently, behaviorally-relevant functional coupling between the SUM and the HPC was shown when SUM spiking became HPC theta phase-modulated particularly before choice points in a continuous alternation task (Ito et al., 2018). Specifically, SUM cells became aligned to the later phases of the CA1 theta cycle, and SUM spiking began close to the time of firing by CA1 interneurons. In the same study, the SUM was demonstrated to be a critical coordinator of communication within the HPC-mPFC-nu. reuniens memory circuit. Thus, it was suggested that the SUM (and by extension, the LHb) dynamically coordinates HPC-related memory circuits by varying spiking relative to HPC theta (Ito et al., 2018).

Not only does the SUM regulate intra- and extra-HPC information processing, but recently it was elegantly demonstrated that the SUM may provide and/or facilitate specific types of information processing in the HPC *via* its distinct direct inputs to the dentate gyrus and region CA2 (Vertes and McKenna, 2000). The SUM to dentate gyrus input may signal contextual novelty while the SUM to CA2 input may signal social novelty (Chen S. et al., 2020). Thus, the SUM appears to be not only involved in the generation of the HPC theta, but it is also important for gating contextual and social information processing within the HPC. Further, SUM enables HPC to communicate with partnered mnemonic structures such as the mPFC and the nu. reuniens. Given the multiple ways that the SUM impacts HPC processing, it is not surprising that lesion or reversible inactivation of the SUM has been shown to impair different types of HPC-dependent behaviors as shown in spatial working memory and certain avoidance tasks (Shahidi et al., 2004a, b; Aranda et al., 2006, 2008), and to be activated during times of stress (Wirtshafter et al., 1998; Ito et al., 2009; Choi et al., 2012). Given the strong input from the LHb to the SUM (Kiss et al., 2002), the SUM is a strong candidate for linking functions of the LHb and HPC (as noted by Goutagny et al., 2013). Here we hypothesize more specifically that the theta-rhythmic firing of SUM neurons may be regulated by LHb behavioral/movement state signals, and in this way, LHb output can generate the conditions needed for animals to flexibly respond to changes in task conditions.

The median raphe (MR) has long been studied for its role in emotion and stress regulation (e.g., Graeff et al., 1996; Andrade et al., 2013). Recently (Baker et al., 2015), it was suggested that the MR may also play a critical role in the coordination of communication between the LHb and HPC since the MR receives strong input from the LHb (Quina et al., 2015; Metzger et al., 2017), the MR has strong projections to a broad extent of the HPC (Azmitia and Segal, 1978; Vertes et al., 1999) and the MR has been shown to significantly impact HPC-dependent behaviors and theta/ripple oscillations (Vertes and Kocsis, 1997; Wang et al., 2015) Thus, the MR is strategically situated to at least assist in the transformation of LHb signals to regulate HPC theta in a manner that facilitates response flexibility. Indeed, a link between serotonergic function and response flexibility is often discussed given the numerous studies that report alterations of serotonin receptors or neurotransmitter release results in difficulties performing classic response flexibility tasks such as set-shifting and strategy shifting (excellent reviews include Nilsson et al., 2015; Alvarez et al., 2021).

**Figure 2:**
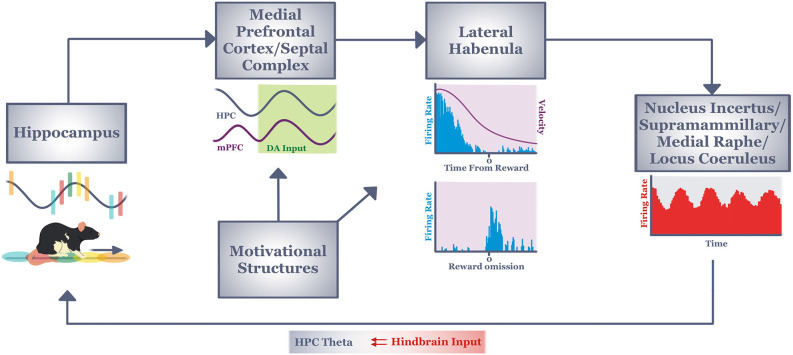
Informationflow through our hypothesized circuit (see text) that mediatescontext-dependent response flexibility during active navigation.Context information from the hippocampus (in the form of place field sequences) informs decision processes of the medial prefrontal cortex and septal complex *via* reward-modulated theta coherence. The output of this memory/decision system integrates with motivational information in the lateral habenula. Lateral habenula neurons integrate this information over many seconds to discharge tonically (to encode current behavioral state, or velocity) or phasically (to encode short duration stimuli/events such as reward outcome). Both types of signals are driven by motivational, memory and decision processing (see text). The output of lateral habenula is hypothesized to provide a signal to hindbrain regions known to regulate hippocampal theta. In this way, the lateral habenula may establish/maintain a neural state in hippocampus that encourages the increased attention and exploratory tendencies needed for animals to respond flexibly to changing task conditions. DA, dopamine; HPC, hippocampus; mPFC, medial prefrontal cortex.

The locus coeruleus (LC) is another hindbrain structure that (in anesthetized and awake rats) is known to project to HPC to impact theta and gamma oscillations (Gray et al., 1975; Walling et al., 2011; Broncel et al., 2021), and to receive at least modest input from the LHb (Mathis et al., 2021). As reviewed by Sara (2009) and Poe et al. (2020), LC neural firing has long been observed to occur in response to novelty, to signal-mismatches events, and to attentional shifts (Aston-Jones and Bloom, 1981a, b; Aston-Jones et al., 1991; Sara and Segal, 1991; Hervè-Minivielle and Sara, 1995; Bouret and Sara, 2005). Our cumulative understanding of the broad impact of the LC across many brain regions as well as the diverse cell types and patterns of LC neural activity have led to theories that especially phasic LC activity enhances the execution of actions in response to unexpected stimuli (Aston-Jones and Cohen, 2005). LC activation, then, effectively resets neural networks to enable rapid switches of cognitive representations or response strategies when needed (Sara and Bouret, 2012). These types of LC responses appear necessary for animals to exhibit adaptive behaviors (Yu and Dayan, 2005). Thus, in addition to the NI, SUM, and MR, the LC is a strong region of interest for its putative role in transmitting LHb signals to the HPC by establishing an HPC theta state that supports response and cognitive flexibility (Figure 2).

## LHb Contributes to Response Flexibility Along Multiple Timescales: Implications for Understanding LHb-Related Psychopathologies

### Multiple Time Scales of LHb Neural Responses and the Impact on Response Flexibility

Based on our extant knowledge and theories about how the LHb contributes to response flexibility, an important and fundamental question arises: How does one reconcile findings that the LHb signals short duration aversive events (Matsumoto and Hikosaka, 2007), while also signaling longer-duration behavioral/movement states (Baker et al., 2015). One explanation is that while LHb broadly tracks the current sensory/behavioral situation, LHb neurons exhibit different patterns of firing depending on the types of information being tracked. LHb neurons may fire phasically to single, short-duration events such as sensory stimuli or a particular goal outcome, or they may fire tonically during longer-duration behavioral state conditions such as movement through space. This sort of dual-coding is not uncommon in the brain. One example is that the same VTA dopamine neurons fire phasically to rewards, and tonically when moving through space (e.g., Puryear et al., 2010; Jo et al., 2013). Either short- or long-duration firing could impact response flexibility that is enabled by the LHb efferent structures described earlier. For example, encountering an unexpected reward should result in phasic LHb cell firing, which would signal efferent structures to increase HPC theta to resolve a potential conflict between expected and actual context features (Mizumori et al., 1999). When tracking sensory/behavioral information across this relatively short period, the LHb may be serving as a brake signal to halt the initiation of potential inappropriate behaviors thereby allowing other adaptive behaviors to commence (Sleezer et al., 2021). When faced with other aversive situations, LHb output may instead result in the engagement of avoidance behaviors. In either case, LHb activity is considered critical during flexible action selection.

By comparison, tonic LHb neural activity for example during spatially-extended navigation, should signal hindbrain regions to elevate HPC theta for the duration of the translocation in space. Such signaling is postulated (see above) to enable the timely enhancement of neuroplasticity mechanisms needed to evaluate the current context so that memories can be updated in preparation for a future decision. That is, LHb-induced HPC theta may result in greater attention and arousal, along with a stronger neuroplasticity state that provides the basis for more efficient and timely context evaluation. Such context analysis is considered essential in order to then execute adaptive and flexible responses in the future. During real world navigation, the LHb engages in both short (sensory) and intermediate-term (during navigation) behavioral monitoring in the same context as evidenced by LHb neurons that show both reward and velocity-related neural codes (Baker et al., 2015). Such dual time frame coding may not be needed in Pavlovian or operant tasks that do not require animals to move about in a spatially-extended environment, but rather they only require information to be associated over relatively short time scales. Thus, assessing LHb in navigating animals may reveal an extended complement of potential contributions to adaptive responding.

In sum, we propose a) that the LHb contributes to response flexibility by tracking behavior across short (in the tens of ms to seconds range) and intermediate (in the range of seconds to hours or more) time frames. Prolonged disturbances of function in either of these time domains can result in different behavioral disorders and psychopathologies.

### LHb Dysfunction in the Short and Intermediate-Term

LHb responses to short-term aversive or appetitive stimuli are necessary for proper adaptive behavior and do not typically result in behavioral maladaptations (Baker et al., 2015). As shown in many of the experiments described previously, inaccurate stimulus coding results in suboptimal decisions. If LHb encoding of behavioral and movement states are deficient, one would expect impaired and inappropriate activation of HPC theta, which could lead to memory deficits. We also know, based on extensive research on the role of LHb in processing aversive information, the LHb plays a substantial role in fear memories. For instance, in the case of fear conditioning, it is important to learn the association between a specific context and the potential threat. In aversive situations, not only does the LHb respond to the aversive stimulus, but also to the cue predicting the onset of the aversive stimulus (Lecca et al., 2017; Lazaridis et al., 2019; Trusel et al., 2019). It takes merely five trials in order for short-scale synaptic changes to occur in the LHb, likely between the LHb and the RMTg (Wang et al., 2017). Also, inactivation of the LHb following fear conditioning of a context resulted in context memory impairments (Durieux et al., 2020). Furthermore, in the same experiment, the c-fos expression following fear conditioning increased in the HPC, mPFC, as well as the LHb, suggesting an involvement of this circuit in contextual fear memories. Thus, in situations when the LHb does not appropriately function over periods of seconds to minutes to hours, one sees impaired decision making and poor context memory.

### Prolonged LHb Dysfunction

Extended and hyperactive LHb aversive signaling can lead to long-lasting plasticity-related changes, resulting in psychiatric disorders such as depression, schizophrenia, and addiction (Metzger et al., 2021). In support of this theory, studies have shown that cocaine exposure induces increased AMPA receptor expression in excitatory LHb neurons, causing long-term potentiation and hyperexcitability (Maroteaux and Mameli, 2012). Similarly, prolonged exposure to an aversive stimulus, such as a foot shock, results in decreased GABAb receptor expression in excitatory LHb neurons, leading to disinhibition and hyperexcitability of LHb neurons (Lecca et al., 2016). Along with synaptic changes, stress and other environmental factors can induce changes in LHb gene expression (Levinstein et al., 2020). Specifically, expression of genes implicated in the RMTg, and not VTA or DRN, pathway is increased in the LHb following stress. These genetic changes alter the strength of the connections between the LHb and downstream structures, resulting in long-lasting changes in plasticity.

Excessive LHb activity can also lead to impaired motivated behavior and result in the pathophysiology of depression. In fact, the LHb is the only brain region that exhibits consistent hyperactivity in depression (Caldecott-Hazard et al., 1988; Andalman et al., 2019). One of the pathways implicated in the expression of depression-like symptoms is the LHb-RMTg pathway that inhibits dopaminergic activity in the VTA and other structures (Proulx et al., 2018). Another pathway underlying this disorder is the LHb’s increased excitation of raphe inhibitory interneuron-based inhibition of serotonergic neurons, which causes a passive coping transition, a marker of depression (Amat et al., 2001; Andalman et al., 2019; Coffey et al., 2020). Ketamine, a common antidepressant medication, has been shown to elevate raphe activity in addition to decreasing the hyperactivity of the LHb, consistent with the role of this pathway in depression (Cui et al., 2018; Yang et al., 2018; López-Gil et al., 2019).

Maladaptive activity in the PFC is frequently tied to the phenotype of schizophrenia (Weinberger et al., 2001). Specifically, the established dopamine hypothesis of schizophrenia posits that schizophrenia arises as a result of dopaminergic hyperactivity in subcortical areas due to cortical dysfunction (Winterer and Weinberger, 2004). In a rat model of schizophrenia, Li et al. (2019) found that there was significant hypofunctionality in the LHb, potentially disinhibiting subcortical dopamine activity. In the same experiment, lesioning the LHb of schizophrenic rats resulted in a significant decrease in cortical activity. Interestingly, functional connectivity between the habenula and cortex increases in schizophrenic patients, suggesting that the habenula may be contributing to the cortical dysfunction seen in schizophrenia (Zhang et al., 2017). Moreover, serotonergic activity in the DRN increases in the pathophysiological profile of schizophrenia. Typically, in healthy brains, the LHb functions to inhibit serotonergic activity in the DRN. However, in schizophrenia, LHb activity is hypoactive, disinhibiting serotonergic activity in the DRN. When the LHb is lesioned in schizophrenic rats, serotonin levels in the DRN, as well as in the mPFC, increased (Li et al., 2019). Owing to these findings, hypofunction in the LHb may contribute to the pathophysiology of schizophrenia.

Likely as a consequence of its critical role in reward valuation and processing, the LHb plays a large role in addiction. Repeated drug exposure results in addiction, or excessive drug-seeking behavior, and is tied to increases in LHb activity (Zhang et al., 2005). Although the LHb processes both aversive and rewarding properties of stimuli (Matsumoto and Hikosaka, 2009b), it appears that the LHb primarily responds to the aversive properties of drug exposure (Zhang et al., 2013). In particular, LHb activity is thought to underlie the negative effects of drug-seeking behavior through its projection to the RMTg (Meye et al., 2015). In support of this finding, Maroteaux and Mameli (2012) showed that cocaine exposure results in increased AMPA receptor expression in LHb neurons selectively projecting to the RMTg. Lastly, chronic drug exposure causes neurodegeneration in the main output fiber bundle of the LHb, the fasciculus retroflexus, disrupting the LHb’s communication with and modulation of downstream monoaminergic structures (Lax et al., 2013).

### Therapeutic Treatment for LHb Dysfunction

Reasoning that long-term LHb function underlies maladaptive behavior, clinicians recently target the LHb in an attempt to correct LHb dysfunction as a therapeutic approach. Deep brain stimulation (DBS) of the LHb has been one such therapeutic procedure that is producing encouraging results (Sartorius et al., 2010). Interestingly, DBS frequency parameters exhibit differential therapeutic outcomes in distinct disorders such as depression and addiction (Ferraro et al., 1996). For an in-depth discussion of DBS in the LHb, see the excellent review by Germann et al. (2021). Relatedly, ketamine has proven to be a successful therapeutic treatment that inhibits the LHb and disinhibits reward centers such as dopamine and serotonin (Yang et al., 2018).

**Figure 3:**
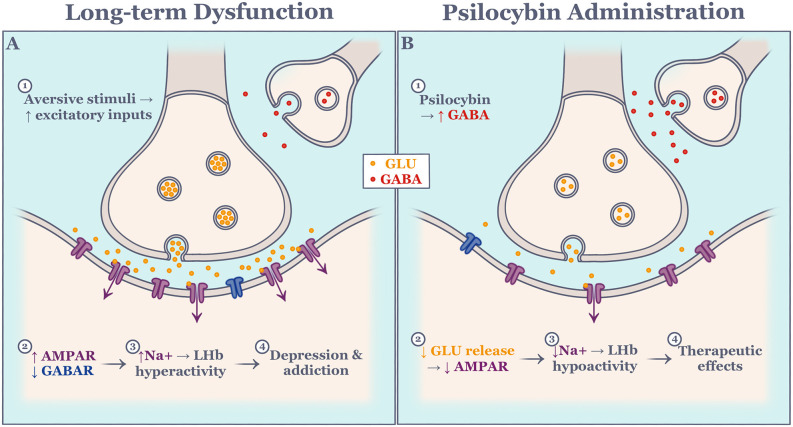
Schematic of hypothesized molecular effect on excitatory lateral habenula neurons during long-term dysfunction **(A)** and psilocybin administration **(B)**. Events are numbered in succession. AMPAR, alpha-amino-3-hydroxy-5-methyl-4-isoxazole propionic acid receptor; GABA, gamma-aminobutyric acid; GABAR, gamma-aminobutyric acid receptor; GLU, glutamate; LHb, lateral habenula; Na, sodium.

Another emerging therapeutic approach for the development of treatments for LHb-related psychopathology focuses on the serotonergic system. Evidence suggests that serotonergic dysfunction results in the inability to adapt to changing environmental conditions, as seen in depression and addiction. Interestingly, the effects of serotonin depletion or overexpression do not always resemble one another across studies. For instance, Lapiz-Bluhm et al. (2009) showed that chronically stressed rats that were serotonin-depleted using *para*-chlorophenylalanine showed significant deficits in reversal learning which was rescued with a serotonin reuptake inhibitor. In a separate study, humans were exposed to a rapid tryptophan depletion paradigm on a reversal-learning task and exhibited slightly improved decision-making (Talbot et al., 2006). Such discrepancy in the literature may be attributed to inter-species differences, as well as the reliability of the drug cocktail protocol. Recent evidence has exposed distinct functions of serotonin subtypes as the main culprit (Alvarez et al., 2021). Importantly, Morris et al. (1999) showed that selective serotonin depletion causes significant increases in LHb activity, mimicking the neural correlate of depression, compared to other structures. Furthermore, tryptophan depletion causes a significant impairment in context memory in mice, implicating the serotonergic system in both motivation and context memory (Uchida et al., 2007). Serotonin 2A receptors subtype has been extensively studied in relation to its influence on behavioral flexibility. A selective serotonin 2A receptor blockade significantly impaired performance on a spatial reversal learning task (Boulougouris et al., 2008). In the same study, a selective serotonin 2C receptor blockade improved performance on a spatial reversal learning task. Interestingly, serotonin 2C and serotonin 2A receptors are found on GABAergic dorsal raphe neurons (Serrats et al., 2005). Psilocybin, being studied as an antidepressant, is a strong serotonin 2A receptor agonist and serotonin 2A receptors are highly expressed in the LHb. This raises the question as to whether psilocybin has modulatory effects on LHb activity. Indeed, activation of serotonin 2A receptors inhibits excitatory LHb neurons, likely through the facilitation of GABAergic transmission, suggesting that psilocybin mimics a neuromodulator and works to rescue LHb dysfunction (Figure 3; Shabel et al., 2012; Metzger et al., 2017). In support of this, psilocybin administration has proven effective in drug-resistant depression (Carhart-Harris et al., 2018), and addiction (Johnson et al., 2014). Whether the therapeutic effects of psilocybin result from the direct action on LHb activity, however, is not yet clear and is in need of further study.

The diversity of LHb inputs from cortical and subcortical areas makes the LHb a prime region for integrating information related to context memory and motivation. Likewise, the LHb’s downstream control of monoaminergic centers, as well as the HPC, implicates it in a number of psychiatric disorders, such as those described above. The reviewed data provide a compelling argument that the LHb should be one of the primary targets of therapeutic intervention, such as with psilocybin, for psychiatric disorders that manifest in context memory, motivational impairments, and certain disorders of behavioral control.

## Conclusion

An outstanding and challenging question is how the LHb enables response flexibility. Here, it is hypothesized that LHb may enable response flexibility by integrating context memory and internal state information to provide critical feedback to memory systems (e.g., the hippocampus) about the outcome of choices and the status of behaviors (e.g., movement velocity). Importantly, this feedback may upregulate neural states in HPC when a context change requires flexible responding to maintain accurate decisions. The upregulation of at least HPC theta could enable the greater attention, arousal, and behavioral activation needed for response flexibility. This feedback system to the HPC, then, may represent a critical step in the loop of information processing between context memory and decision systems, intrinsic motivational systems, response implementation, and memory updating and retrieval that is needed to flexibly redirect responses. Supporting our hypothesis, improper functioning of the LHb results in impairments in behavior related to response flexibility such as those seen in psychiatric disorders.

## Data Availability Statement

Publicly available datasets were analyzed in this study. This data can be found here: https://www.mizumorilab.com/.

## Author Contributions

VH and SM contributed to the conceptualization and writing of this review. All authors contributed to the article and approved the submitted version.

## Conflict of Interest

The authors declare that the research was conducted in the absence of any commercial or financial relationships that could be construed as a potential conflict of interest.

## Publisher’s Note

All claims expressed in this article are solely those of the authors and do not necessarily represent those of their affiliated organizations, or those of the publisher, the editors and the reviewers. Any product that may be evaluated in this article, or claim that may be made by its manufacturer, is not guaranteed or endorsed by the publisher.
